# Epistatic interaction between *PKD2* and *ABCG2* influences the pathogenesis of hyperuricemia and gout

**DOI:** 10.1186/s41065-020-0116-6

**Published:** 2020-01-27

**Authors:** Zheng Dong, Jingru Zhou, Shuai Jiang, Yuan Li, Dongbao Zhao, Chengde Yang, Yanyun Ma, Hongjun He, Hengdong Ji, Li Jin, Hejian Zou, Jiucun Wang

**Affiliations:** 1 State Key Laboratory of Genetic Engineering, Collaborative Innovation Center for Genetics and Development, School of Life Sciences, Fudan University Jiangwan Campus, 2005 Songhu Road, Shanghai, 200438 People’s Republic of China; 2 Division of Rheumatology and Immunology, Changhai Hospital, Shanghai, China; 3 Division of Rheumatology, Ruijin Hospital, Shanghai Jiaotong University School of Medicine, Shanghai, China; 4 Division of Rheumatology, Taixing People’s Hospital, Jiangsu Province, China; 5 Division of Rheumatology, Taizhou People’s Hospital, Jiangsu Province, China; 6 Fudan-Taizhou Institute of Health Sciences, Taizhou, Jiangsu Province China; 7 Division of Rheumatology, Huashan Hospital, Fudan University, 12 Wulumuqi Zhong Road, Shanghai, 200040 People’s Republic of China; 8 Institute of Rheumatology, Immunology and Allergy, Fudan University, Shanghai, China

**Keywords:** Epistasis, Serum urate, Body mass index (BMI), Gene transcript, Gender, Enhancer

## Abstract

**Background:**

Genetic background affects serum urate concentration and gout risk, especially regarding these variants in the urate-transporter gene *ABCG2*. However, the role of epistasis between *PKD2* and *ABCG2* on the pathogenesis of gout is poorly understood. Here we assess this epistatic interaction in the progression from elevated serum urate to gout.

**Results:**

We identified two epistatic interaction pairs (rs2728121: rs1481012 and rs2728121: rs2231137) were associated with urate levels in 4914 Chinese individuals (*P*_int_ = 0.018 and 0.004, respectively). Using subgroup analysis for gender and BMI, we found the degree of associations was varied by gender and BMI. The SNP pair rs2728121:rs1481012 influenced urate levels in females and overweight subjects (*P*_int_ = 0.006 and 0.022, respectively), but rs2728121:rs2231137 did in males, overweight and normal-weight subjects (*P*_int_ = 0.017, 0.047 and 0.013, respectively). Consistent results were also observed in associations between these epistatic interactions with hyperuricemia. Next, the SNP pair rs2728121:rs2231137 was identified to influence the development of gout from both hyperuricemia and healthy (*P*_int_ = 0.035 and 0.001, respectively), especially in males (*P*_int_ = 0.030 and 0.001, respectively). Furthermore, we demonstrated that interacting regions were enriched by regulatory elements. Finally, we observed a strong gene co-expression pattern between *PKD2* and *ABCG2* (r = 0.743, *P* = 5.83E-06).

**Conclusion:**

Our findings indicate epistasis between *PKD2* and *ABCG2* influence serum urate concentrations, hyperuricemia and gout risk, thus providing insight into the pathogenesis of gout.

## Background

Urate is the end product of purineger breakdown in humans. Elevated urate levels in the blood (hyperuricemia) can cause the deposition of monosodium urate (MSU) crystals in the joints and tissues that play a predominant role in the development of gout [1–5]. Gout is a form of acute inflammatory arthritigers [6, 7] that affects range from 0.1% to approximately 10% of individuals worldwide [8]. Although hyperuricemia can cause gout [9], not all hyperuricemia patients develop gout [10]. Generally, only a quarter of hyperuricemia patients develop gout, suggesting that hyperuricemia is necessary but not sufficient for gout development [10]. Several loci in different genes have been identified to affect serum urate concentration and gout risk [1, 10]. However, these loci only explained ~ 7% of the variance in urate levels [1, 4]. Therefore, it is necessary to identify other genetic factors contribute to the progression from elevated serum urate to hyperuricemia to gout.

*PKD2* is located nearby *ABCG2*, which encodes a urate transporter that plays a certain role in serum urate concentrations and gout risk [4, 11]. Even though serval GWAS studies found *PKD2* variants associated with serum urate and gout, none variants in the *PKD2* gene were independently associated with serum urate and gout conditional on the *ABCG2* variants [1–3, 12]. Based on that, *PKD2* is commonly not considered as a candidate gene for serum urate and gout. This also happened for *WDR1*, which is adjacent to a urate transporter gene *SLC2A9*. Although one studies reported no relationship between *SCL2A9* variants and hyperuricemia/gout [13], a number of GWAS and functional studies revealed a significant association between *SCL2A9* variants and hyperuricemia/gout [1–4, 12]. A previous study showed that epistatic interactions between *WDR1* and *SLC2A9* regulated serum urate concentrations, suggesting the biological value of epistatic interactions in the pathogenesis of gout [14].. Therefore, we attempted to investigate a potential epistatic interaction between *PKD2* and *ABCG2*.

Here we aimed to explore epistasis between *PKD2* and *ABCG2* using a four-step approach. First, the epistatic effect of loci on serum urate was investigated. Next, these identified urate-related epistatic interactions were tested in the development of hyperuricemia and gout, separately. Common heterogeneity factors, including gender, body mass index (BMI) and smoking status, were considered as covariates in the analysis. Besides, enrichment analysis was performed to provide insight into the biological function of these urate-related epistatic interactions. Finally, the interplay between *PDK2* and *ABCG2* transcripts was investigated. All statistical analyses were performed with adjustment for marginal effects. Through this strategy, our results suggested that epistatic interactions between *PDK2* and *ABCG2* contributed to serum urate and gout and that *PKD2* is supposed to influence the progression from elevated serum urate to hyperuricemia to gout by epistatically interacting with *ABCG2*.

## Results

### Epistatic interactions between *PDK2* and *ABCG2* affected serum urate concentrations

Four SNP pairs were tested in our study (Table 1). Of them, two SNP pairs (rs2728121:rs1481012 and rs2728121:rs2231137) were found to influence serum urate concentrations with contradictory effects (Estimate = − 14.487, *P*_int_ = 0.018 and Estimate = 9.781, *P*_int_ = 0.004, respectively) (Table 1). The two SNP pairs rs2728121:rs1481012 and rs2728121:rs2231137 could explain 0.099 and 0.164% of urate variance without conditioning on the marginal SNPs, separately (Table 1).

**Table 1:** Associations between serum urate and epistatic interactions in *PKD2* and *ABCG2*

Chr.	SNP_1_	Pos_1_	A_1_	SNP_2_	Pos_2_	A_2_	Dist	LD (*r*^2^)		Estimate	*P*_int_	Variance explained (%)
4	rs2725215	88,961,571	T	rs1481012	89,039,082	G	77,511	0.605	Male	−2.309	0.693	–
									Female	−12.817	0.236	–
									Total	−7.020	0.181	–
4	rs2725215	88,961,571	T	rs2231137	89,061,114	T	99,543	0.116	Male	4.830	0.618	–
									Female	20.178	0.220	–
									Total	9.306	0.270	–
4	rs2728121	88,997,102	C	rs1481012	89,039,082	G	41,980	0.370	Male	−6.051	0.379	–
									Female	−33.315	0.006	0.493
									Total	−14.487	0.018	0.099
4	rs2728121	88,997,102	C	rs2231137	89,061,114	T	64,012	0.045	Male	8.980	0.017	0.144
									Female	9.250	0.171	–
									Total	9.781	0.004	0.164

Chr, chromosome of an SNP pair. SNP_1_ (SNP_2_), Pos_1_ (Pos_2_), name and position of the first (second) SNP. A_1_, effect allele of SNP_1_; A_2_, effect allele of SNP_2_. dist, distance in bp between two SNPs. LD (*r*^2^), linkage disequilibrium between two SNPs. *P*_int_, *P*-value of the interaction between SNP pair in serum urate was calculated by linear regression adjusted age, gender, and target SNPs. Variance explained of the interaction between the SNP pair in serum urate was calculated by linear regression

Next, our results showed that these associations were significantly modified by gender (Table 1). SNP pair rs2728121:rs2231137 only affected urate (Estimate = 8.980, *P*_int_ = 0.017) in the male subgroup. In contrast, SNP pair rs2728121:rs1481012 only contributed to the concentrations of serum urate in females (Estimate = − 33.315, *P*_int_ = 0.006). Lager urate variance (0.493%) could be explained by epistatic interactions rs2728121:rs1481012 in female subgroup than in all subjects, highlighting the role of gender on associations between these epistasis and serum urate concentrations.

### Epistatic interactions of *PDK2* and *ABCG2* affected the development of hyperuricemia and gout

Given that elevated serum urate is a critical risk factor for the development of hyperuricemia and gout, we further assessed the contributions of the two identified urate-related epistatic interactions on hyperuricemia and gout risk. Regarding hyperuricemia, SNP pair rs2728121:rs2231137 significantly increased hyperuricemia risk (Estimate = 0.193, *P*_int_ = 0.009). In essence, SNP pair rs2728121:rs2231137 only affected the risk of hyperuricemia in males (Estimate = 0.206, *P*_int_ = 0.018), but did not in females (*P*_int_ = 0.427). In contrast, SNP pair rs2728121:rs1481012 decreased the risk of hyperuricemia in females (Estimate = − 0.648, *P*_int_ = 0.017) (Table 2), but did not in males (*P*_int_ = 0.483). All the above findings are pretty consistent with the results in serum urate.

**Table 2:** Associations between urate-related epistatic interactions and hyperuricemia/gout

SNP_1_	SNP_2_		Hyperuricemia Vs. Control			Gout Vs. Hyperuricemia			Gout Vs. Control		
			Estimate	SE	*P*_int_	Estimate	SE	*P*_int_	Estimate	SE	*P*_int_
rs2728121	rs1481012	Male	−0.108	0.153	0.483	0.188	0.245	0.444	0.077	0.258	0.765
		Female	−0.648	0.272	0.017	−0.528	0.947	0.577	−0.897	0.835	0.282
		Total	−0.254	0.130	0.051	0.165	0.234	0.482	−0.004	0.239	0.987
rs2728121	rs2231137	Male	0.206	0.087	0.018	0.338	0.156	0.030	0.524	0.161	0.001
		Female	0.118	0.148	0.427	0.100	0.536	0.852	0.229	0.523	0.661
		Total	0.193	0.074	0.009	0.313	0.149	0.035	0.480	0.149	0.001

SNP_1_ (SNP_2_), name of the first (second) SNP. SE, standard error. *P*_int_, *P*-value of the interaction between the SNP pair in hyperuricemia and gout were calculated by logistic regression adjusted age, gender, and target SNPs

For the development from hyperuricemia to gout, SNP pair rs2728121:rs2231137 played an important role (Estimate = 0.313, *P*_int_ = 0.035), especially in males (Estimate = 0.338, *P*_int_ = 0.030) (Table 2). However, SNP pair rs2728121:rs1481012 did not contribute to this progression in neither males nor females (*P*_int_ = 0.444 and *P*_int_ = 0.577, respectively). Because SNP pair rs2728121:rs2231137 influence all progressions from elevated serum urate to gout, it had a definitive effect in the pathogenesis of gout (Estimate = 0.480, *P*_int_ = 0.001), especially regarding in males (Estimate = 0.524, *P*_int_ = 0.001).

### Associations between epistatic interactions and serum urate in BMI and smoking subgroups

Our previous studies have suggested that body mass index (BMI) and cigarette smoking could modify urate levels [4, 9, 15]. But their impact on the degree of associations between epistasis and serum urate was not determined. Therefore, we further performed subgroup analyses for BMI and smoking status in our study.

For the subgroups of BMI, SNP pair rs2728121:rs2231137 was identified to be associated with urate in normal (Estimate = 11.456, *P*_int_ = 0.013) and overweight individuals (Estimate = 9.844, *P*_int_ = 0.047) (Table 3). SNP pair rs2728121:rs1481012 only affected the concentrations of serum urate in overweight subgroup (Estimate = − 21.702, *P*_int_ = 0.022). However, no significant associations were observed in any subgroups of smoking status (all *P*_int_ > 0.05) (Table 3).

**Table 3:** Associations between epistatic interactions and serum urate in subgroups of BMI and smoking status

SNP_1_	SNP_2_		Subgroup-1			Subgroup-2			Subgroup-3		
			Estimate	SE	*P*_int_	Estimate	SE	*P*_int_	Estimate	SE	*P*_int_
rs2728121	rs1481012	BMI	19.794	41.811	0.637	−10.483	8.306	0.207	−21.702	9.477	0.022
		Smoke	−25.467	15.210	0.095	−7.751	10.478	0.460	−9.196	8.343	0.271
rs2728121	rs2231137	BMI	24.512	22.948	0.288	11.456	4.620	0.013	9.844	4.962	0.047
		Smoke	9.972	8.385	0.235	7.106	5.223	0.174	7.592	4.608	0.100

SNP_1_ (SNP_2_), name of the first (second) SNP. SE, standard error. *P*_int_, *P*-value of the interaction between the SNP pair in serum urate was calculated by linear regression adjusted age, gender and target SNPs. Subgroup of BMI: 1, Underweight (BMI < 18.5); 2, Normal (18.5 ≦ BMI < 25); 3, Overweight (BMI ≧ 25). Subgroup of smoke: 1, non-smokers; 2, former smokers; 3, current smokers

### Colocation of interacting regions with regulatory elements

By annotating urate-related interacting regions with chromatin state, we found several strong and weak enhancers were located at these regions, especially at gene *PKD2* (Additional file 1: Fig. S1 and Additional file 2: Figure S2). Consistent with the above result, transcription factors and DNase binding sites, were also observed at the *PKD2* gene region (Additional file 1: Figure S1 and Additional file 2: Figure S2). Our findings supposed the potential regulatory effects across these interacting regions, especially in *PKD2*. Besides, *ABCG2* has been identified as a urate transporter in previous studies that played a key role in serum urate and gout [1, 11, 16]. Above all, we hypothesized that *PKD2* can influence serum urate by epistatically interacting with *ABCG2*.

To further explore potential regulatory effects at the *PKD2* region, two sets of SNPs were tested using enhancer enrichment analysis (Table 4). Regarding urate-related SNPs identified in a recent genome-wide association study [1], we found a significant enrichment in enhancer regions in aHuvec cell line (3.6-fold enrichment, *P* = 0.012), especially for strong enhancers (5.7-fold enrichment, *P* = 0.005) (Table 4). For all loci in *PKD2*, distinct enrichment for these loci in enhancers were observed across various cell lines (H1, HepG2, Huvec, HSMM, NHLF, HMEC, GM12878, and NHEK cell lines) (Table 4). Our findings provided a piece of supporting evidence that regulatory factors in *PKD2* control the concentrations of serum urate by epistatic interactions with *ABCG2*.

**Table 4:** Enhancer enrichment analysis of loci in *PKD2*

Set	Cell type		All enhancers				Strongest enhancers			
	ID	Description	Obs	Exp	Fold	*P*	Obs	Exp	Fold	*P*
1	Huvec	umbilical vein endothelial cells	5	1.4	3.6	0.012	4	0.7	5.7	0.005
2	H1	H1 cell line	4	10.5	0.4	0.994	4	0.9	4.5	0.013
	HepG2	hepatocelluar carcinoma	24	9.3	2.6	2.50E-05	24	3.1	7.8	< 1.0E-06
	Huvec	umbilical vein endothelial cells	41	11.4	3.6	< 1.0E-06	29	5.7	5.1	< 1.0E-06
	HSMM	skeletal muscle myoblasts	30	12.9	2.3	1.80E-05	4	5.7	0.7	0.818
	NHLF	lung fibrolasts	43	11.6	3.7	< 1.0E-06	15	4.4	3.4	4.20E-05
	HMEC	mammary epithelial cells	36	15	2.4	1.00E-06	10	5.7	1.7	0.065
	GM12878	B-lymohocyte, lymphoblastoid	20	11.8	1.7	0.016	13	4.3	3	4.36E-04
	NHEK	epidermal keratinocytes	30	13	2.3	2.00E-05	17	5.7	3	7.40E-05

Set one was urate-related SNPs identified in a recent genome-wide association study. Set two was all SNPs in the gene region of *PKD2*

### The interplay between *PDK2* and *ABCG2* transcripts

**Fig. 1:**
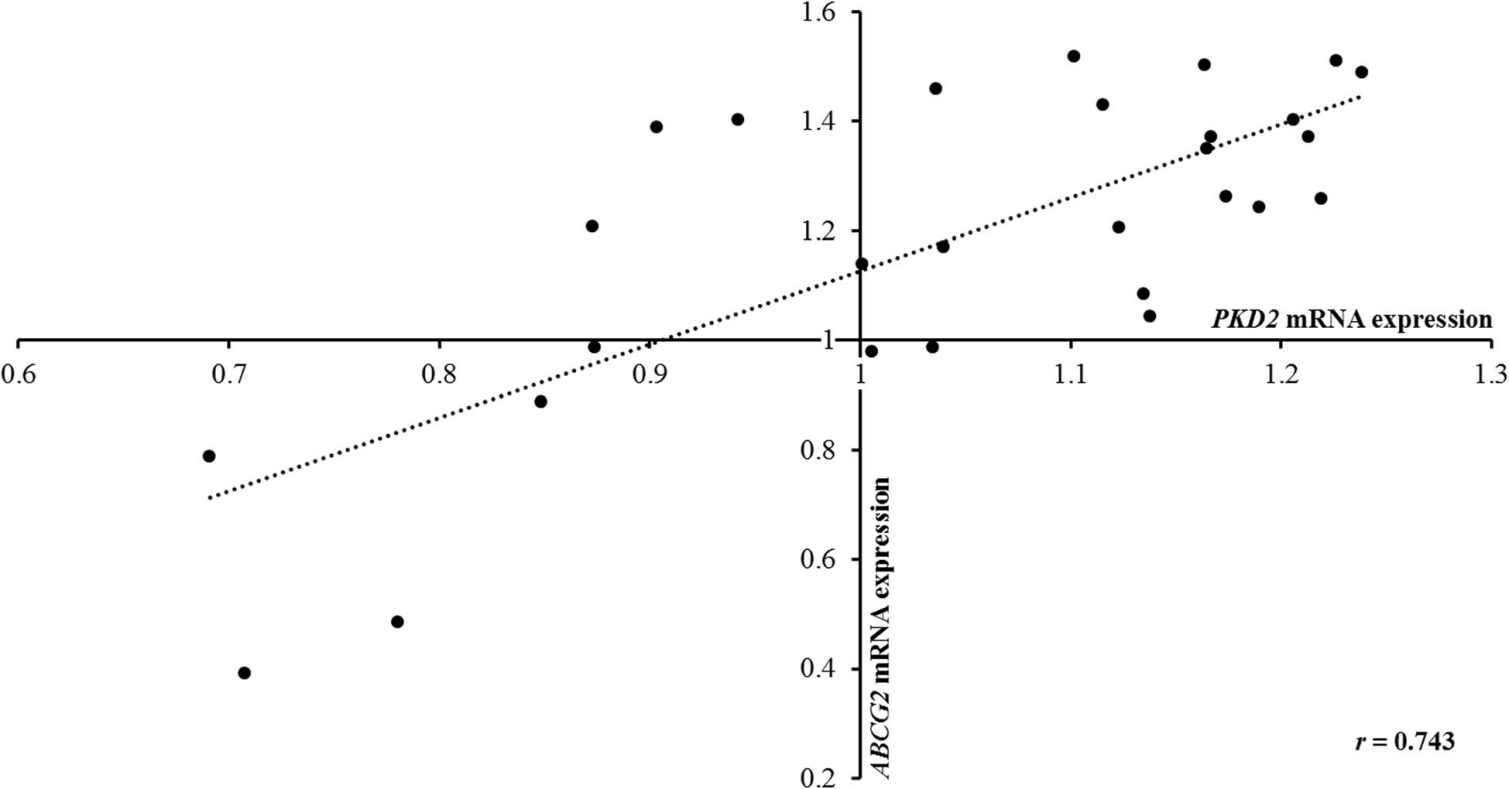
The gene co-expression pattern between *PDK2* and *ABCG2*

To further confirm these urate-related epistatic interactions between *PKD2* and *ABCG2*, gene transcripts of *PKD2* and *ABCG2* were investigated. The result indicated a strong positive correlation between *PKD2* with *ABCG2* transcripts (r = 0.743, *P* = 5.83E-06) (Fig. 1), thus supporting our hypothesis that *PKD2* can affect serum urate by epistatically interacting with *ABCG2*.

## Discussion

Our study explored the effect of epistatic interactions between *PKD2* and *ABCG2* on the progression from elevated serum urate to gout. Here we identified two SNP pairs (rs2728121:rs1481012 and rs2728121:rs2231137) significantly associated with serum urate, hyperuricemia or gout. The role of these two identified urate-related epistatic interactions was investigated in detail, including subgroup analyses for heterogeneity factors (such as gender, BMI and smoking status), enrichment analysis and gene co-expression analysis.

*ABCG2* gene encodes a urate transporter that influences serum urate concentrations and gout risk. It has been proved to be one of the strongest risk factors for the development of gout [1, 11]. However, there are conflicting results regarding the association between *PKD2* and urate/gout. Recent studies suggested that *PKD2* is associated with urate and gout [17], but contrary results were observed in other studies [3]. Furthermore, no functional experiments are suggesting the contribution of *PKD2* on serum urate and gout to date. Therefore, we performed a systemic analysis to explore the role of *PKD2* on the development of hyperuricemia and gout.

Our observations showed that two SNP pairs (rs2728121:rs1481012 and rs2728121:rs2231137) were associated with serum urate concentrations or hyperuricemia risk. SNP pair rs2728121: rs2231137 was also identified to be associated with the development of gout from both hyperuricemia and healthy (Tables 1 and 2). These results revealed a potential mechanism for the biological role of *PKD2* in serum urate and gout. SNP pair rs2728121:rs1481012 can explain 0.099% of urate variance, and even more in females (0.493%). Whereas SNP pair rs2728121: rs2231137 explains 0.164% of the variance (Table 1). These results suggested that epistasis may be an additional way to solve the ‘missing heritability’ problem. Additionally, consistent with our previous results [4, 9, 15], heterogeneity factors, such as gender and BMI, modified the degree of associations between epistatic interactions and serum urate/gout (Table 3).

The potential mechanism of epistatic interactions between *PKD2* and *ABCG2* genes were analyzed by enrichment analysis and gene co-expression analysis. We supposed that *PKD2* can indirectly influence the development of gout, by interacting with *ABCG2* since no direct role of *PKD2* have been identified until now. Enrichment analysis showed that regulatory factors were enriched in *PKD2*, suggesting the potential regulatory function of *PKD2*. To further confirm this result, gene transcripts of these two genes were examined. The result indicated a positive correlation between *PDK2* and *ABCG2* gene expression, further supporting our hypothesis that *PKD2* can influence serum urate by epistatically interacting with *ABCG2*.

## Conclusions

Our results uncover that epistatic interactions between *PKD2* and *ABCG2* influence all progressions from elevated serum urate to gout. Furthermore, the degree of association has been found to vary with gender and BMI.

## Methods and materials

### Study subjects

This study was approved by the Ethical Committees of the School of Life Sciences of Fudan University (approval number of 140) and conducted following the principles of the Declaration of Helsinki. All subjects in this study provided written informed consent. All 582 gout patients enrolled in this study were diagnosed with gout (OMIM: #138900) following the American College of Rheumatology diagnostic criteria [18]. These gout patients did not use any urate-lowering drugs for two weeks before sample collection. Their clinical information was collected at Changhai Hospital, Taixing People’s Hospital, and Taizhou People’s Hospital.

Furthermore, 4332 individuals without a history of gout were enrolled from the Taizhou Longitudinal Study [19]. Among them, 1387 subjects were considered hyperuricemia patients due to their high serum urate levels (> 417 umol/L) [20]. The other individuals were treated as healthy controls. All 4332 subjects were divided into subgroups according to their body mass index (BMI) and smoking status recorded in questionnaires. Three BMI subgroups (underweight: BMI < 18.5; normal-weight: 18.50 ≤ BMI < 25; overweight: BMI ≥ 25) were defined following the categories of the World Health Organization (WHO) [4, 15]. Besides, smoking status categories included non-smoker, former smoker, and current smoker. The detailed characteristics of these participants were illustrated in Additional file 3: Table S1.

### Target loci selected in *ABCG2*

Here we leveraged *ABCG2* SNPs rs2231137 and rs1481012 in our analysis. The SNP rs2231137 is a missense variant that strongly linked with serum urate and gout [1, 4]. Another SNP rs1481012 is strongly LD with rs2231142 (r^2^ = 1 in Eastern Asian), which is a significant gout-related loci [11, 21]. Due to the complexity of the sequence region for rs2231142, it was difficult to detect its genotype by using SNaPshot (TianHao, China). Therefore, we defined rs1481012 as a replacement of rs2231142 in our further analysis. Besides, the two SNPs are independent of each other (r^2^ = 0.186 in Eastern Asian) and other *ABCG2* SNPs highly LD (r^2^ > 0.6) with the two loci were also excluded. Finally, the two SNPs are common variants in the Chinese population with a minor allele frequency (MAF) > 0.2. Altogether, we selected the two SNPs in our study.

### SNP genotype and real-time qPCR

Peripheral blood was collected from all participants in this study. DNA was extracted from blood samples according to standard procedures. To measure gene expression, we randomly collected RNA samples from 58 male subjects. These subjects were a subset of 2945 healthy individuals enrolled in genotype analysis. Detailed information for DNA and RNA processing have been described in our previous study [4]. Genotyping of target SNPs in *PKD2* (rs2725215 and rs2728121) and *ABCG2* (rs2231137 and rs1481012) was performed by SNaPshot (TianHao, China). Gene transcript was tested by real-time qPCR.

### Enrichment analysis

Two sets of loci in *PKD2* were used in our enrichment analysis. One was comprised of urate-related SNPs identified in a recent genome-wide association study [1]. Another contained all SNPs in the gene region of *PKD2*. We performed cell-type-specific enhancer enrichment analysis in both two SNP sets using HaploReg (http://archive.broadinstitute.org/mammals/haploreg/haploreg.php) [22]. For each set of SNPs, the overlap of SNPs with ENCODE [23] cell-type-specific enhancers was compared to all 1000 Genomes variants with a minor allele frequency above 5%. The fold enrichment and *P*-value were calculated using a binomial test. SNPnexus [24] and UCSC genome browser [25] was used to annotate these loci. Enlight (http://enlight.wglab.org) was applied to draw gene-regional plots and show epigenetic modifications in *PKD2* [26]. Additionally, linkage disequilibrium (LD) relationships between target SNPs were analyzed using SNAP (http://archive.broadinstitute.org/mpg/snap/index.php) [27].

### Statistical analysis

*P*-values for associations between target SNP pairs with serum urate concentrations were calculated by linear regression with adjustment for age, gender, and marginal effects. The variance of serum urate explained by each SNP pair was calculated by linear regression. All *P*-values for associations between SNP pairs with hyperuricemia and gout were measured by logistic regression adjusted for age, gender and marginal effects. Subgroup analyses for gender, BMI and smoking status were also performed in our study. Moreover, the relationship between *PKD2* and *ABCG2* transcripts was investigated. *P* values of < 0.05 were considered statistically significant. All statistical analyses in this study were processed using R (Version 3.0.2: www.r-project.org/).

## Supplementary information

**Additional file 1: Figure S1.** Chromatin state analysis of *PKD2* and *ABCG2* genes by Enlight.

**Additional file 2: Figure S2.** Chromatin state analysis of PKD2 and ABCG2 genes by the UCSC genome browser.

**Additional file 3: Table S1.** Characteristics of all participants in our study. HUA, hyperuricemia. The data are shown as the mean (SD).

## Data Availability

The datasets used and/or analysis in the current study can be obtained from the corresponding author according to reasonable requirements.

## Abbreviations

*ABCG2*: ATP-binding cassette subfamily G member 2
BMI: Body mass index
LD: Linkage disequilibrium
MSU: Monosodium urate
OMIM: Online Mendelian Inheritance in Man
OR: Odds ratio
*P*_int_: *P* values for epistatic interactions
*PKD2*: Polycystin-2
RNA: Ribonucleic acid
*SLC2A9*: Solute Carrier Family 2 Member 9
SNP: Single nucleotide polymorphism
*WDR1*: WD Repeat Domain 1
WHO: World Health Organization
