# Supplementary material for: Epistatic interaction between PKD2 and ABCG2 influences the pathogenesis of hyperuricemia and gout
Source: Hereditas. 2020 Jan 27;157:2. doi: 10.1186/s41065-020-0116-6 (PMC6986014; doi:10.1186/s41065-020-0116-6)
Supplement: Supplementary file 1 — Additional file 1: Figure S1. Chromatin state analysis of PKD2 and ABCG2 genes by Enlight. [file 41065_2020_116_MOESM1_ESM.pdf]

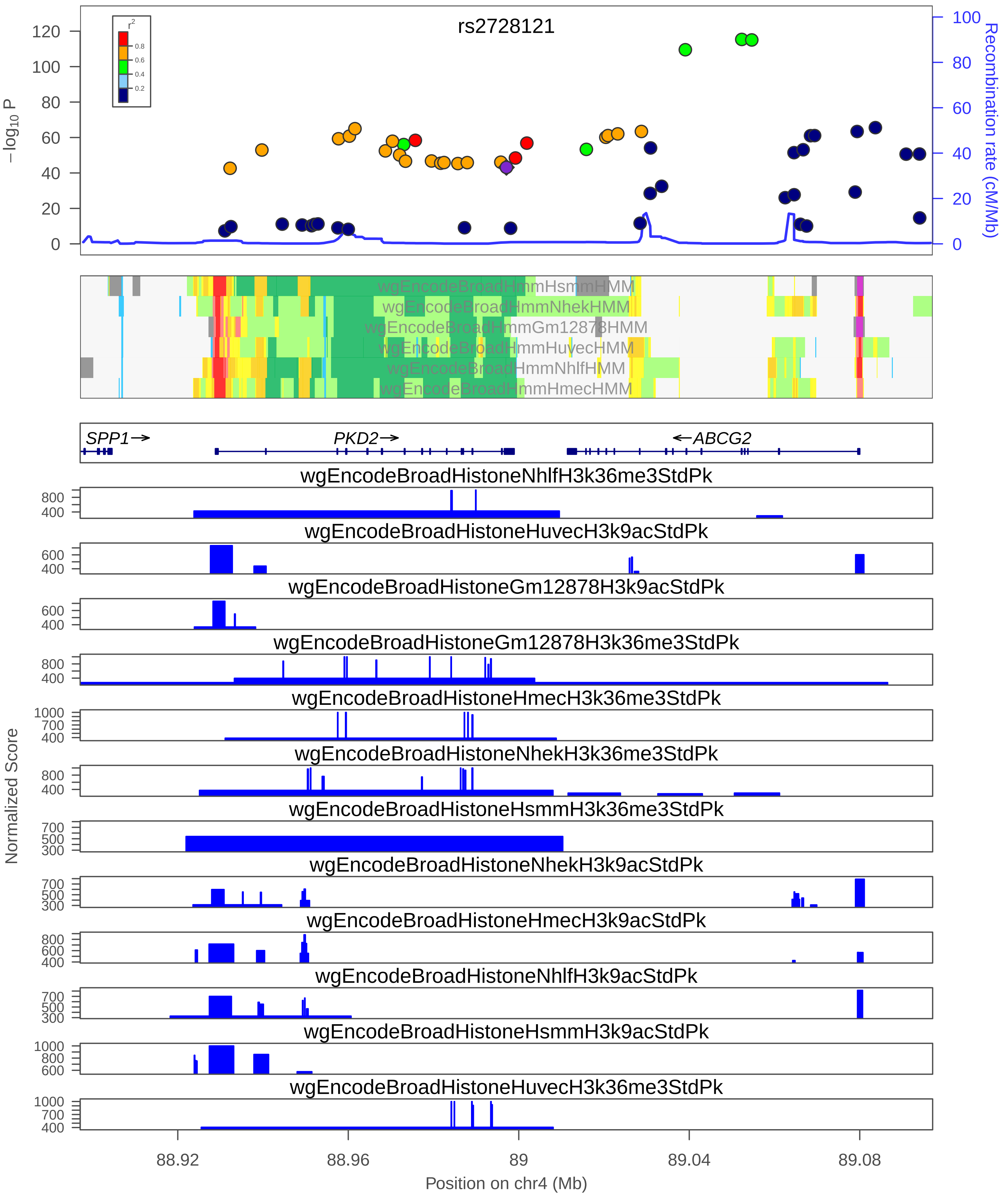
rs2728121
r2
0.8
0.6
0.4
0.2
−log10 P
Recombination rate (cM/Mb)
wgEncodeBroadHmmHsmmHMM
wgEncodeBroadHmmNhekHMM
wgEncodeBroadHmmGm12878HMM
wgEncodeBroadHmmHuvecHMM
wgEncodeBroadHmmNhlfHMM
wgEncodeBroadHmmHmecHMM
SPP1→
PKD2→
←ABCG2
wgEncodeBroadHistoneNhlfH3k36me3StdPk
wgEncodeBroadHistoneHuvecH3k9acStdPk
wgEncodeBroadHistoneGm12878H3k9acStdPk
wgEncodeBroadHistoneGm12878H3k36me3StdPk
wgEncodeBroadHistoneHmecH3k36me3StdPk
wgEncodeBroadHistoneNhekH3k36me3StdPk
wgEncodeBroadHistoneHsmmH3k36me3StdPk
wgEncodeBroadHistoneNhekH3k9acStdPk
wgEncodeBroadHistoneHmecH3k9acStdPk
wgEncodeBroadHistoneNhlfH3k9acStdPk
wgEncodeBroadHistoneHsmmH3k9acStdPk
wgEncodeBroadHistoneHuvecH3k36me3StdPk
Normalized Score
88.92
88.96
89
89.04
89.08
Position on chr4 (Mb)

Category Plot Legend

- Active Promoter
- Weak Promoter
- Inactive/poised Promoter
- Strong enhancer
- Weak/poised enhancer
- Insulator
- Transcriptional transition/elongation
- Weak transcribed
- Polycomb–repressed
- Heterochromatin; low signal; Repetitive/Copy Number Variation
