# Supplementary figures and images for: Epistatic interaction between PKD2 and ABCG2 influences the pathogenesis of hyperuricemia and gout
Source: Hereditas. 2020 Jan 27;157:2. doi: 10.1186/s41065-020-0116-6 (PMC6986014; doi:10.1186/s41065-020-0116-6)

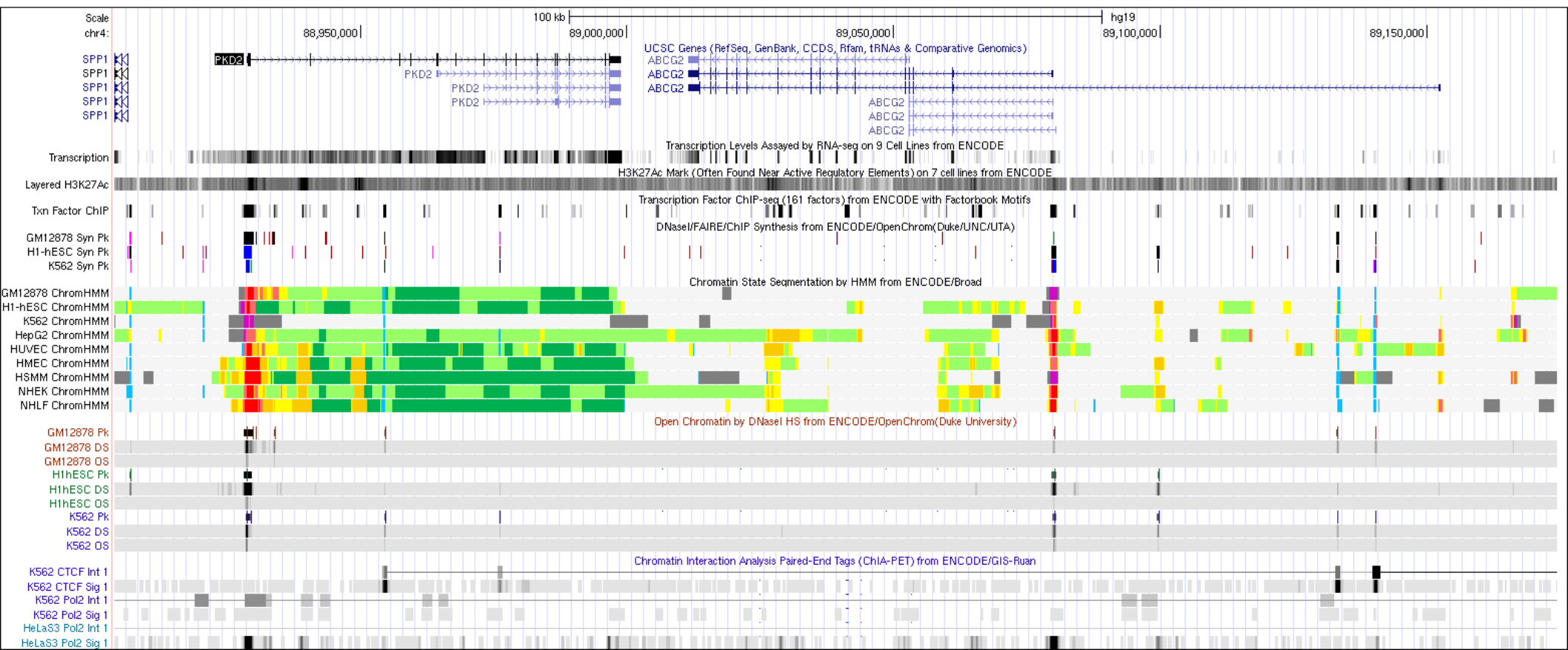

Supplement: Supplementary file 2 — Additional file 2: Figure S2. Chromatin state analysis of PKD2 and ABCG2 genes by the UCSC genome browser. [file 41065_2020_116_MOESM2_ESM.pdf]
