# Supplementary material for: Epistatic interaction between PKD2 and ABCG2 influences the pathogenesis of hyperuricemia and gout
Source: Hereditas. 2020 Jan 27;157:2. doi: 10.1186/s41065-020-0116-6 (PMC6986014; doi:10.1186/s41065-020-0116-6)
Supplement: Supplementary file 3 — Additional file 3: Table S1. Characteristics of all participants in our study. HUA, hyperuricemia. The data are shown as the mean (SD). [file 41065_2020_116_MOESM3_ESM.pdf]

S.Table 1. Characteristics of all participants in our study

Characteristics	Control	HUA
Number	2945	1387
Male (%)	70.53%	76.42%
Age	69.00 (9.25)	67.91 (11.93)
Height (cm)	161.37 (7.52)	162.63 (7.54)
Weight (kg)	63.71 (9.96)	68.56 (11.12)
BMI	24.43 (3.24)	25.88 (3.52)
Serum urate (umol/l)	300.73 (63.13)	482.30 (61.14)
Total bilirubin (umol/l)	19.39 (8.39)	18.98 (7.59)
Glucose (mmol/L)	5.60 (1.67)	5.59 (1.31)
Cholesterol (mmol/L)	4.75 (0.93)	5.06 (1.52)
Triglyceride (mmol/L)	1.51 (0.96)	2.23 (1.76)
Creatinine (umol/L)	71.75 (17.61)	89.80 (29.24)
Blood urea nitrogen (mmol/L)	5.48 (2.13)	6.24 (1.95)

HUA, hyperuricemia. Data is shown as mean (SD).
